# Chronic pancreatitis of the pancreatic remnant is an independent risk factor for pancreatic fistula after distal pancreatectomy

**DOI:** 10.1186/1471-2482-14-54

**Published:** 2014-08-15

**Authors:** Marius Distler, Stephan Kersting, Felix Rückert, Peggy Kross, Hans-Detlev Saeger, Jürgen Weitz, Robert Grützmann

**Affiliations:** 1 Department of General, Thoracic and Vascular Surgery, University Hospital Carl Gustav Carus, TU, Dresden, Germany; 2 Department of Surgery, University Hospital Mannheim, Mannheim, Germany

**Keywords:** Distal pancreatectomy, Pancreatic fistula, Chronic pancreatitis, Pancreas surgery

## Abstract

**Background:**

There is an ongoing debate about the best closure technique after distal pancreatectomy (DP). The aim of the closure is to prevent the formation of a clinically relevant post-operative pancreatic fistula (POPF). Stapler technique seems to be equal compared with hand-sewn closure of the remnant. For both techniques, a fistula rate of approximately 30% has been reported.

**Methods:**

We retrospectively analyzed our DPs between 01/2000 and 12/2010. In all cases, the pancreatic duct was over sewn with a separately stitched ligation of the pancreatic duct (5*0 PDS) followed by a single-stitched hand-sewn closure of the residual pancreatic gland. The POPF was classified according to the criteria of the International Study Group for Pancreatic Fistula (ISGPF). Univariate and multivariate analyses of potential risk factors for the formation of POPF were performed. Indications for operations included cystic tumors (n = 53), neuroendocrine tumors (n = 27), adenocarcinoma (n = 22), chronic pancreatitis (n = 9), metastasis (n = 6), and others (n = 7).

**Results:**

During the period, we performed 124 DPs (♀ = 74, ♂ = 50). The mean age was 57.5 years (18–82). The POPF rates according to the ISGPF criteria were: no fistula, 54.8% (n = 68); grade A, 24.2% (n = 30); grade B, 19.3% (n = 24); and grade C, 1.7% (n = 2). Therefore, in 21.0% (n = 26) of the cases, a clinically relevant pancreatic fistula occurred. The mean postoperative stay was significantly higher after grade B/C fistula (26.3 days) compared with no fistula/grade A fistula (13.7 days) (p < 0.05). The uni- and multivariate analyses showed chronic pancreatitis of the pancreatic remnant to be an independent risk factor for the development of POPF (p = 0.004 OR 7.09).

**Conclusion:**

By using a standardized hand-sewn closure technique of the pancreatic remnant after DP with separately stitched ligation of the pancreatic duct, a comparably low fistula rate can be achieved. Signs of chronic pancreatitis of the pancreatic remnant may represent a risk factor for the development of a pancreatic fistula after DP and therefore an anastomosis of the remnant to the intestine should be considered.

## Background

A pancreatic resection left of the superior mesenteric artery (SMA) is termed left resection or distal pancreatectomy (DP). This pancreatic resection technique can be performed, according to the indication of the operation, as a spleen-preserving resection, or it can be combined with a splenectomy. There are various indications for a distal pancreatectomy. The most common indications are patients with malignant diseases, cystic or neuroendocrine tumors or even chronic pancreatitis [1]. A major cause of postoperative morbidity is the development of a pancreatic fistula (POPF). A pancreatic fistula after DP can lead to hemorrhages, abscesses, sepsis or wound infections [2]. A variety of procedures have been recommended to reduce the frequency of pancreatic fistula [2,3]. In particular, the stapler transection and the hand-sewn closure technique have been widely analyzed. For both techniques, a fistula rate of approximately 30% has been reported [4,5]. The present analysis demonstrates our experience with the hand-sewn closure technique (including separately stitched ligation of the pancreatic duct) at a single German pancreatic center. The primary outcome of our study was the formation of a pancreatic fistula. Furthermore, we analyzed our data regarding the risk factors for the development of a POPF.

## Methods

### Patients

Between January 2000 and December 2010 we performed a total of 872 pancreatic resections in the Department for General, Thoracic and Vascular Surgery in the Carl Gustav Carus University Hospital, Dresden. Eight pancreato-biliary surgeons performed 124 consecutive DPs in this period. Indications for operations were cystic tumors (mainly IPMN) or suspicion of a malignant pancreatic tumor (or proven malignant tumor) or metastasis. Postoperative histological diagnosis after pancreatic left resections were IPMN (n = 53), neuroendocrine tumors (n = 27), adenocarcinoma (n = 22), chronic pancreatitis (n = 9), metastasis (n = 6), and others (n = 7). The patient characteristics are summarized in Table 1.

**Table 1:** Demographic and clinical data from our patient cohort (n = 124)

	**Distal pancreatectomies (n = 124)**
**Sex (m/f)**	n = 74 (59.7%)/n = 50 (40.3%)
**Age y (±SD)**	57.5 (±14.2) 95% CI 55.0-60.1
**BMI (m/kg**^**2**^**)**	25.8 (±5.1) 95% CI 24.9-26.8
**ASA scores**	
**1**	n = 14 (11.3%)
**2**	n = 75 (60.5%)
**3**	n = 35 (28.2%)
**Nicotine abuse (yes/no)**	n = 27 (21.8%)/n = 97 (78.2%)
**Alcohol abuse (yes/no)**	n = 35 (28.2%)/n = 89 (71.8%)
**Hypertension (yes/no)**	n = 56 (45.2%)/n = 68 (54.8%)
**Weight loss (yes/no)**	n = 38 (30.6%)/n = 86 (69.4%)
**Preoperative Diabetes (Total)**	n = 32 (25.8%)
**Oral**	n = 22 (17.7%)
**IDDM**	n = 10 (8.0%)
**Diagnosis**	
**Adenocarcinomas**	n = 22 (17.7%)
**IPMN**	n = 53 (42.7%)
**Chronic pancreatitis**	n = 9 (7.3%)
**Metastasis**	n = 6 (4.8%)
**NET**	n = 27 (21.9%)
**Others**	n = 7 (5.6%)
**Postoperative Pancreatic Fistula**	
**(POPF)**	
**Grade A**	n = 30 (24.2%)
**Grade B**	n = 24 (19.3%)
**Grade C**	n = 2 (1.7%)
**OP-time (min) (±SD)**	282.2 (±106.8) 95% CI 263.2-301.2
**Intraop. blood loss (ml) (±SD)**	834 (±787.1) 95% CI 694.6-1217.4

### Data collection

The medical records from a prospective database of patients who underwent DP were analyzed retrospectively for each case. Multivisceral resections had not been included into this cohort to obtain a homogenous group. In accordance with the guidelines for human subject research, approval was obtained from the ethics committee at the Carl Gustav Carus University Hospital. The survey data were complemented by the clinical notes of the patients’ physicians and surgeons. Furthermore, the histological diagnostic findings of each case were reviewed. In particular, pathology reports of each patient were screened for histological signs of chronic pancreatitis in the frozen section of the pancreatic remnant, independently from the major pathological finding (indication for operation). Information for possibly deceased patients was obtained from family members or from the general practitioner. The median postoperative follow up of all patients was 27 months. The clinical data and demographics of our cohort are shown in Table 1.

For the statistical analysis, we used the following parameters: sex, age, intraoperative blood loss, OR time, diagnosis, ASA scores, nicotine abuse, alcohol abuse, hypertension, pre- and/or postoperative diabetes, insulin use, weight loss, histologically proven chronic pancreatitis of the pancreatic remnant and UICC stage (if available) (Table 2). The postoperative events and clinical outcomes were also recorded prospectively and analyzed retrospectively (Table 1).

**Table 2:** Univariate analysis of independent risk factors for the development of a clinically relevant POPF after DP (Type 0/A versus B/C)

	**POPF 0/A n = 98**	**POPF B/C n = 26**	**P-value**
**Sex (m/f)**	**n = 59/n = 39**	**n = 15/n = 11**	0.816*
**Age**	95% CI 0.952-1.009		0.179** (OR 0.98)
**Blood loss (intraoperative)**	95% CI 1.000-1.001		0.007** (OR 1.001)
**OR time**	95% CI 1.000-1.007		0.082** (OR 1.003)
**Diagnosis**			0.072*
PDAC	n = 16	n = 6	
IPMN	n = 42	n = 11	
Chronic pancreatitis	n = 8	n = 1	
Metastasis	n = 4	n = 2	
NET	n = 25	n = 2	
Others	n = 3	n = 4	
**ASA score**			0.335*
1	n = 9	n = 5	
2	n = 60	n = 15	
3	n = 29	n = 6	
**Nicotine**			
Yes/No	n = 20/n = 78	n = 7/n = 19	0.474*
**Alcohol abuse**			
Yes/No	n = 26/n = 72	n = 9/n = 17	0.416*
**Hypertension**			
Yes/No	n = 48/n = 50	n = 8/n = 18	0.097*
**Preoperative diabetes**			
Yes/No	n = 28/n = 70	n = 4/n = 22	0.172*
**Postoperative diabetes**			
Yes/No	n = 29/n = 69	n = 4/n = 22	0.145*
**Preoperative IDDM**			
Yes/No	n = 10/n = 88	n = 0/n = 26	0.089*
**Chronic pancreatitis in remnant**			
Yes/No	n = 12/n = 86	n = 9/n = 17	0.007*
**Preoperative weight loss**			
Yes/No	n = 31/n = 67	n = 7/n = 19	0.643*
**UICC Stage (any malignant tumor)**			
0	n = 69	n = 16	
Ia	n = 3	n = 0	
Ib	n = 6	n = 1	0.512*
IIa	n = 4	n = 2	
IIb	n = 12	n = 6	
III	n = 1	n = 1	
IV	n = 3	n = 0	

*χ2-test , **binary logistic regression analysis; p < 0.05 statistically significant.

### Hand-sewn closure technique of the pancreatic remnant

**Figure 1:**
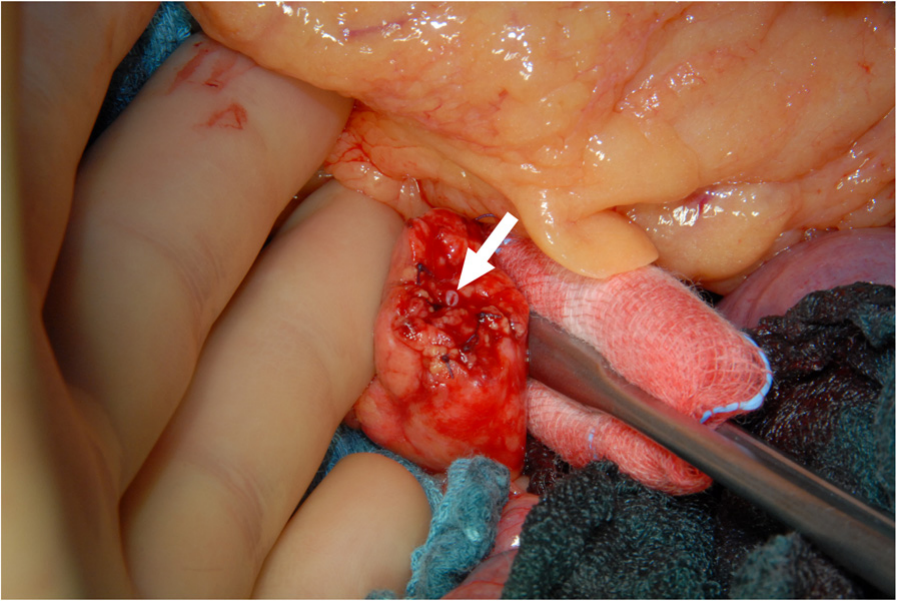
Pancreatic remnant after transection with a scalpel and stitch ligation (5*0 Vicryl) of small vessels. Arrow: pancreatic duct.

**Figure 2:**
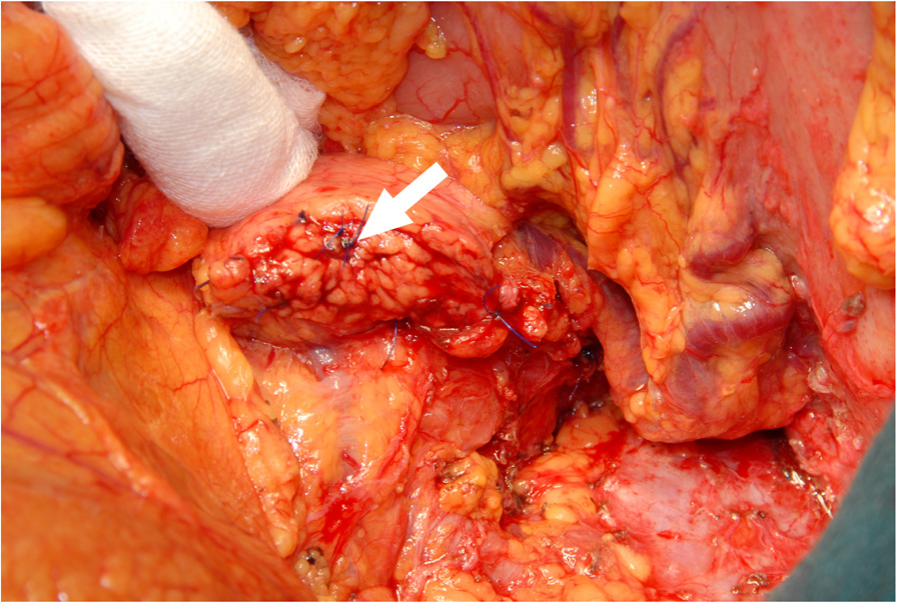
Arrow: Figure-of-eight stitch ligation of the pancreatic duct (5*0 PDS).

**Figure 3:**
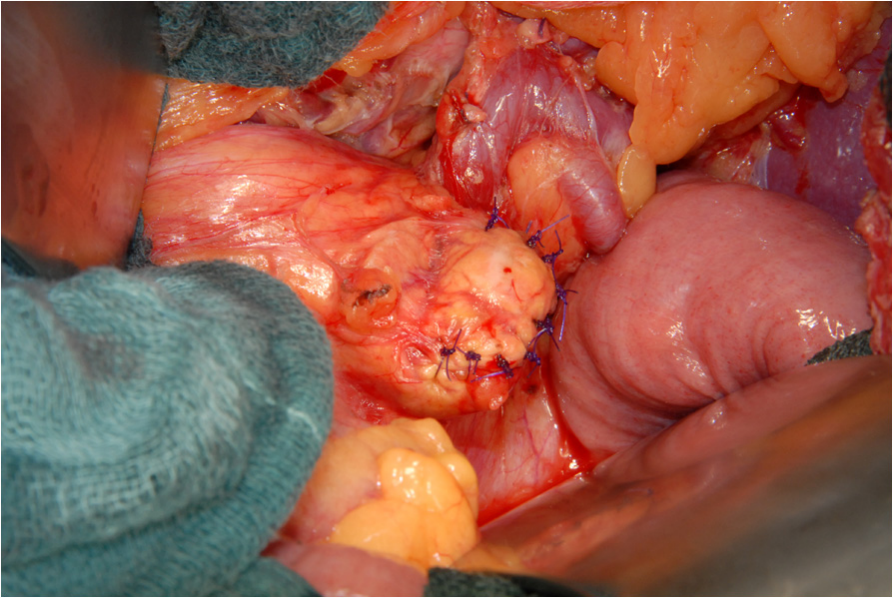
Simple, single-stich suture fish-mouth closure of the pancreatic remnant (4*0 PDS).

All resections were performed as open operations. In all cases with malignancy, a concomitant splenectomy was performed. In benign cases, the operation was performed as a spleen-preserving operation, if possible. The pancreatic gland was cut wedge-shaped to facilitate a fish-mouth closure of the stump.In all cases, the pancreatic duct was thoroughly identified and over sewn with a separately stitched figure-of-eight ligation (5*0 PDS) (Figures 1 and 2). Then, a simple single-stitched hand-sewn closure of the fish-mouth was accomplished. In Figures 1,2,3 our technique is shown in detail. No additional treatment (e.g. octreotide) or covering of the pancreatic remnant was performed. Every patient received one intraabdominal drain, and the enzyme parameter (amylase) was determined daily in the fluid collected from the drain after the operation.

### Definitions

Postoperative Pancreatic Fistula (POPF) was defined analogous to the ISGPF criteria. By examination of the fluid collected from the intraoperatively placed drain, a POPF was defined as amylase content greater than three times the upper normal serum level after postoperative day three. The POPF was stratified into grades A, B and C [6].

### Management of the persistent pancreatic fistula

We did not use any standard treatment protocol for POPF. In general, the drains were left in place, or in cases with drain removal, the drains were re-placed interventionally. In some cases, octreotide was also used. Usually, oral food intake was not stopped. Therefore, the basic principle of therapy was adequate drainage and patience.

### Statistical analysis

The statistical analysis was performed using SPSS for Windows, version 19.0 (SPSS, Inc., Chicago, IL). All clinical and pathological characteristics were grouped to build categorical or nominal variables. The cutoff points used for categorizations were based on previously described cutoff points in the literature and/or recursive partitioning as previously described [7]. Continuous data are presented as 95% confidence intervals (95% CI) and SDs. Univariate examination of the relationship between the assessed criteria and POPF was performed with a χ2-test and by binary logistic regression analysis. For multivariate analysis a logistic regression model of the preoperative parameters with stepwise backwards elimination based on likelihood ratios was employed to test for independent predictors of POPF. However the odds ratios of the parameters OR time, age and blood loss were close to 1 and therefore they rendered the model unstable, these parameters were not included into the multivariate analysis. A two-sided p-value <0.05 was considered statistically significant.

## Results

### Patient demographics and preoperative parameters

From 2000 to 2010, we performed 124 DPs. The mean age of the patients undergoing DP was 57.5 years, with a range of 18 to 82 years, and there were 74 female and 50 male patients. The indications for the operation are shown in Table 1. Approximately 60% of the patients had an ASA II status, and the mean BMI was 28.5. The most frequent concomitant diagnoses at the time of presentation were hypertension (45.2%), diabetes (25.8%), nicotine abuse (18.1%) and alcohol abuse (28.2%). The mean operation time was 282 minutes (range 100–640), and the mean intraoperative blood loss was 834 ml (range 0–2500) (Table 1).

### Postoperative pancreatic fistula (POPF)

By using the ISGPF grading system, we identified a total of 56 POPFs (45.2%). We recognized 30 (24.2%) grade A fistulas and 24 (19.3%) grade B fistulas, and 2 (1.7%) patients developed a grade C fistula (Table 1). Thus in 21% of the cases, a clinically relevant POPF (grade B/C) occurred. Repeat operations were performed on both patients with grade C fistulas. In one case, an intraabdominal abscess was drained, and an additional drain was placed. In the second patient, the pancreatic remnant was closed by a pancreatojejunostomy (end-to-side). Two patients (1.6%) died during the hospital stay, but none of them died due to a POPF. One patient died after bleeding of esophageal varicose, and the other died due to a complication after pleural drainage. The mean postoperative stay was significantly higher after grade B/C fistulas (26.3 days) compared with no fistula/grade A fistulas (13.7 days) (p < 0.001). In addition, there were no differences between the eight pancreatic surgeons performing DP.

### Univariate and multivariate analysis of risk factors for POPF

The above mentioned perioperative parameters and patient demographics were used for univariate analysis regarding the development of a clinically relevant POPF (B/C). Univariate analysis showed that histologically proven chronic pancreatitis of the pancreatic remnant (p = 0.007) and intraoperative blood loss (p = 0.007, OR 1.0) are a risk factor for the development of a POPF (Table 2). The diagnosis alone (p = 0.072), preoperative IDDM (p = 0.089), OR time (P = 0.082) and the presence of hypertension (p = 0.097) appear to be marginally significant factors (Table 2). A multivariate analysis with backward elimination confirmed the results of the univariate analysis. Chronic pancreatitis of the pancreatic remnant is an independent risk factor for the development of a clinically relevant POPF (grade B/C) (p = 0.004, OR 7.09). Furthermore, the presence of hypertension was detected as a significant factor for POPF (p = 0.05, OR 2.80) (Table 3).

**Table 3:** Multivariate analysis of risk factors for the development of a clinically relevant POPF

		**95% confidence interval OR**		
	**OR**	**Lower**	**Upper**	**P-value**
**Step 1**				
Sex	0.60	0.131	2.802	0.520
PDAC	0.50	0.084	2.984	0.447
IPMN	0.04	0.002	1.003	0.050
Chronic pancreatitis	1.38	0.113	16.939	0.798
Metastasis	0.28	0.031	2.540	0.259
NET	4.68	0.423	51.818	0.208
ASA	0.85	0.302	2.421	0.768
Nicotine	2.34	0.525	10.446	0.265
Alcohol	1.82	0.504	6.572	0.361
Hypertension	2.32	0.609	8.854	0.218
Preoperative Diabetes	0.93	0.160	5.490	0.943
Postoperative Diabetes	0.33	0.054	2.077	0.240
Chronic pancreatitis in remnant	6.51	1.390	30.545	0.017
Preoperative Weight loss	0.40	0.101	1.648	0.208
**Step 13**				
Hypertension	2.80	0.980	8.033	0.050
Chronic pancreatitis in remnant	7.09	1.867	26.994	0.004

## Discussion

The technique of stump closure after DP has been widely debated. Different approaches have been tested in various studies, and in a comparative analysis, no significant advantage for any specific method was detected [8]. Recent publications, such as the DISPACT trial, have compared the stapler and the hand-sewn closure techniques. No difference between these techniques was shown in regard to POPF [4]. Moreover a current randomized controlled trial by Carter et al. evaluated the additional effect of fibrin glue or autologous falciform patch after stapler or hand- sewn closure of the pancreatic remnant [5]. They found no benefit for this additional measure regarding POPF. Finally, various studies evaluated the use of somatostatin analogues perioperatively or postoperatively. However, a meta-analysis by Koti et al. found a decrease of morbidity only in selected patients [9]. Thus, POPF remains a major source of morbidity and is therefore a relevant clinical problem after DP even though it does not lead to a high postoperative mortality. Considering this background, we present our experiences with the hand-sewn closure technique in the current report.

In our study, we specifically focused on factors contributing to pancreatic fistula. In the present series, all DPs were performed during open laparotomy using the same technique of selective ligation of the pancreatic duct. Currently, there is a tendency to perform this operation laparoscopically. This approach, however, presents the same complications as those associated with open resection and stapler closure; safe closure of the pancreatic remnant is therefore an important issue for laparoscopic distal pancreatectomy [10].

During the follow up, nearly half of the patients (45%) showed a POPF after PD. However, a clinically relevant POPF (grade B/C) was evaluated in only 21% of the cases. These results are consistent with the published data [5, 11, 12,]. The recently published DISPACT trial showed comparable results related to POPF (approximately 20-21%) for the stapler transection and the hand-sewn closure technique [4]. By using a standardized closure technique, we could reproduce the published results for development of a clinically relevant POPF in our cohort. Although it should be noted that the present study is a retrospective analysis.

We support the importance of the selective ligation of the pancreatic duct. A previous study by Pannegeon et al. [11] confirmed the findings of a multivariate analysis by Bilimoria et al. [12] indicating that specific ligation of the main pancreatic duct is an independent protective factor for POPF. Other authors have noted that an inability to find the main pancreatic duct for ligation is a major factor related to the occurrence of postoperative fistula [13].

In our study, chronic pancreatitis of the pancreatic remnant was identified as an independent risk factor for POPF in the univariate (p = 0.007) and multivariate analyses (OR 7.09) (Table 2 and 3). Similarly to other authors, we believe that this is due to downstream stenosis of the main pancreatic duct, most likely in the pancreatic head region, due to chronic inflammation. Preoperative diabetes was not detected as a significant risk factor for POPF in our analysis, but it is important to note that in most cases, diabetes is concomitant with chronic pancreatitis and therefore could be a preoperative indicator [14]. We infer from our data that in cases with a potential obstruction of the pancreatic duct in the pancreatic head/periampullary region (e.g. due to chronic pancreatitis), and therefore an increased likelihood of POPF, the pancreatic remnant should be anastomosed. This seems especially important in patients with preoperatively or intraoperatively detected dilatation of the pancreatic duct. Pancreatic anastomosis seems to be a safe method of closure in these cases after DP. However, one could speculate that pancreatic leakage following small bowel anastomosis could result in potentially more serious complications than leakage without anastomosis (e.g. activation of pancreatic enzymes, bacterial contamination). Therefore, a pancreatic anastomosis following DP should only be performed if it can be carried out safely [15].

Hypertension has also been noted as a risk factor for the development of POPF in our analyses. This might be due to the pathophysiological effect of hypertension, which causes generalized atherosclerosis and thereby limits the microcirculation of the tissue. Perfusion is a particularly important factor for wound healing. By compromising the healing process, hypertension could negatively affect the postoperative course. This negative effect of hypertension has been reported previously [16]. Moreover, hypertension is also a parameter of the POSSUM score, which accurately predicts morbidity in various operation procedures [17,18].

In addition, our analysis confirms that patients with a clinically relevant pancreatic fistula had a mean extension of their hospital stay (B/C fistula (26.3 days) vs. no fistula/grade A fistula (13.7 days)) (p < 0.001). This tendency was also recently reported by the DISPACT trial [4].

## Conclusion

Our data support the assumption that DP can now be performed without significant mortality (i.e., 1.6% in our analysis) [4,11,18]. However, the morbidity after DP is still high due to the occurrence of POPF. By selective stitched ligation of the pancreatic duct and fish-mouth closure of the pancreatic remnant, a low POPF rate comparable to the results in the literature could be achieved. Chronic inflammation of the pancreatic remnant should be considered a risk factor for POPF during the intraoperative decision process for stump closure, and maybe an anastomosis of the remnant to the small intestine, e.g. using a jejunal limb, should be considered in these cases.

## Abbreviations

POPF: Postoperative pancreatic fistula; DP: Distal pancreatectomy; ISGPF: International Study Group for Pancreatic Fistula; IDDM: Insulin depending diabetes mellitus.

## Competing interests

The authors declare no competing interests. This research received no specific grant from any funding agency in public, commercial, or not-for-profit sectors.

## Authors’ contributions

DM wrote the manuscript, collected the data, interpreted the results and statistically analyzed the data, RF and KS analyzed the data statistically, interpreted the results and critically revised the manuscript, KP collected the data and wrote parts of the manuscript, SHD and JW designed the concept of the manuscript operations and critically revised the manuscript, and GR designed the study, collected data, and drafted the manuscript. All authors read and approved the final manuscript.

## Pre-publication history

The pre-publication history for this paper can be accessed here:

http://www.biomedcentral.com/1471-2482/14/54/prepub
